# Hypertrophy of the Anterior External Arcuate Fasciculus: A Rare Variant With Implications for the Development of the Arcuate Nucleus

**DOI:** 10.3389/fnana.2020.595500

**Published:** 2020-11-27

**Authors:** Renee Stonebridge, Ross J. Taliano, Terra D. Velilla, Douglas C. Anthony

**Affiliations:** ^1^Department of Pathology and Laboratory Medicine, Lifespan Academic Medical Center, Providence, RI, United States; ^2^Department of Pathology and Laboratory Medicine, Warren Alpert Medical School of Brown University, Providence, RI, United States; ^3^Department of Neurology, Warren Alpert Medical School of Brown University, Providence, RI, United States

**Keywords:** arcuate nucleus, medulla, calbindin-2, inferior olivary nucleus, development

## Abstract

A rare anatomic variant of a markedly enlarged anterior external arcuate fasciculus (AEAF) on the ventral medullary surface is reported and compared to two controls. The hypertrophic AEAF was nine times larger in diameter than normal, whereas the arcuate nucleus (AN) and inferior olivary nucleus (ION) appeared histologically normal in size and neuronal distribution, and morphometric analysis of the AN confirmed that it was within the normal range. Calbindin-2 (calretinin, CALB2) expression was identified in the AN and in the fibers of the normal AEAF. The hypertrophic AEAF did not contain calbindin-2–expressing fibers. CALB2 expression was also present in the ventrolateral portion of the ION, both in the index case and in one of the control cases. The origin of the additional fibers was not identified; however, the potential origin of these fibers and its implications for the development of the AEAF are discussed.

## Introduction

The arcuate nucleus (AN) of the medulla has been proposed to play an important role in regulation of respiration in response to increasing levels of carbon dioxide (Filiano et al., 1990; Filiano and Kinney, 1992; Okada et al., 2001; Zec and Kinney, 2003; Rubens and Sarnat, 2013). Located on the ventral surface of the medulla overlying the pyramid, this curved band spans the entire rostrocaudal length of the inferior olivary nucleus (ION) as a discontinuous sheet of neurons (Mikhail and Ahmed, 1975; Filiano and Kinney, 1992; Zec et al., 1997; Gray et al., 2005). The AN is considered a pre-cerebellar nucleus and contains two main clusters of neurons: medial clusters located just medial to the pyramid and lateral clusters lying on the ventral surface of the pyramid (Mikhail and Ahmed, 1975; Filiano and Kinney, 1992; Zec et al., 1997; Gray et al., 2005; Yu et al., 2014). The AN receives its principal input from the cerebral cortex via fibers traveling within or adjacent to the corticospinal tract. There are two main projection pathways for axons from the AN. Some axons from the AN project anteriorly onto the ventral surface of the medulla in the anterior external arcuate fasciculus (AEAF), which traverses the inferior olive as a circumolivary bundle and ultimately projects to the cerebellum via the inferior cerebellar peduncle; others project posteriorly into the medullary reticular formation (Rasmussen and Peyton, 1946; Zec et al., 1997; Fu and Watson, 2012). In the mouse, the cerebellar target of the AN-like structure is the paraflocculus of the contralateral cerebellar cortex (Fu and Watson, 2012). In humans, fibers from the AN project both ventrally in the AEAF and dorsally to the caudal raphe and superficial ventrolateral medullary regions (Zec et al., 1997), but the cerebellar target of the human AN has not yet been fully established. The AN in humans has an average transverse area of 1–3 mm^2^ (Paradiso et al., 2018).

The AEAF is a bundle of axons on the ventral surface of the medulla that arises predominantly from the AN (Rasmussen and Peyton, 1946; Zec et al., 1997; Gray et al., 2005; Fu and Watson, 2012). It begins as a midline fiber bundle and travels along the ventral and lateral subpial surface of the medulla, crosses over the surface of the inferior olive as the circumolivary bundle, and enters the inferior cerebellar peduncle to terminate in the cerebellum, although the exact cerebellar termination has not been fully determined in humans (Rasmussen and Peyton, 1946; Gray et al., 2005; Rubens and Sarnat, 2013). Dye-tracking studies have identified the AEAF as a band of fibers located along the entire extent of the ventrolateral surface of the medulla at the level of the ION that forms an arching bundle of fibers (Zec et al., 1997). Within this bundle are some serotonergic [5-hydroxytryptamine (5-HT)] fibers that extend along the medial and ventral rim of the pyramids (Kinney, 2005; Kinney et al., 2007). These 5-HT fibers, although a minority of the total number of AEAF fibers, arise from 5-HT neurons within the AN, also a minority population in the AN, providing further evidence that the AEAF arises from the AN (Kinney, 2005; Kinney et al., 2007; Paterson et al., 2009; Kinney and Haynes, 2019). There are neither prior descriptions of the size or axonal density of the AEAF, nor any prior descriptions of anatomic abnormalities of the AEAF.

A number of studies of the human AN in sudden unexplained infant death (SUID, also known as sudden infant death syndrome) suggest that the AN is hypoplastic or absent in SUID, raising the possibility that its proposed role in chemoreception, especially for carbon dioxide, may play a role in SUID by depressing hypercapnic respiration (Filiano et al., 1990; Filiano and Kinney, 1992; Zec et al., 1997; Matturri et al., 2000, 2002a,b; Kinney et al., 2002, 2007; Zec and Kinney, 2003; Kinney, 2005; Rubens and Sarnat, 2013; Kinney and Haynes, 2019). In this article, we describe a patient with an abnormally large AEAF and a normal-sized AN. The findings have implications for the development of the AN and its major efferent pathway.

## Description of a Case

A 50 year old woman with a medical history of hypertension, chronic kidney disease, obesity, depression, schizophrenia, and obstructive sleep apnea had a witnessed cardiac arrest at home. When emergency medical assistance arrived, ventricular tachycardia was identified, and cardiopulmonary resuscitation was initiated. Despite full resuscitative measures, the ventricular tachycardia progressed to asystole. Upon arrival at the hospital, the patient remained unresponsive, and continued cardiopulmonary resuscitation efforts failed to regain cardiac rhythm or circulation. The only known neurologic history was an episode 8 months prior to death of severe, frontal headaches and blurred vision lasting for ~4 days and was thought to be the result of uncontrolled hypertension, given that her systolic blood pressure at the time was >200 mmHg. Neuroimaging (computed tomography with contrast) did not demonstrate any abnormality. Her schizophrenia was well-controlled with medications, although compliance with medications was not consistent.

**Figure 1:**
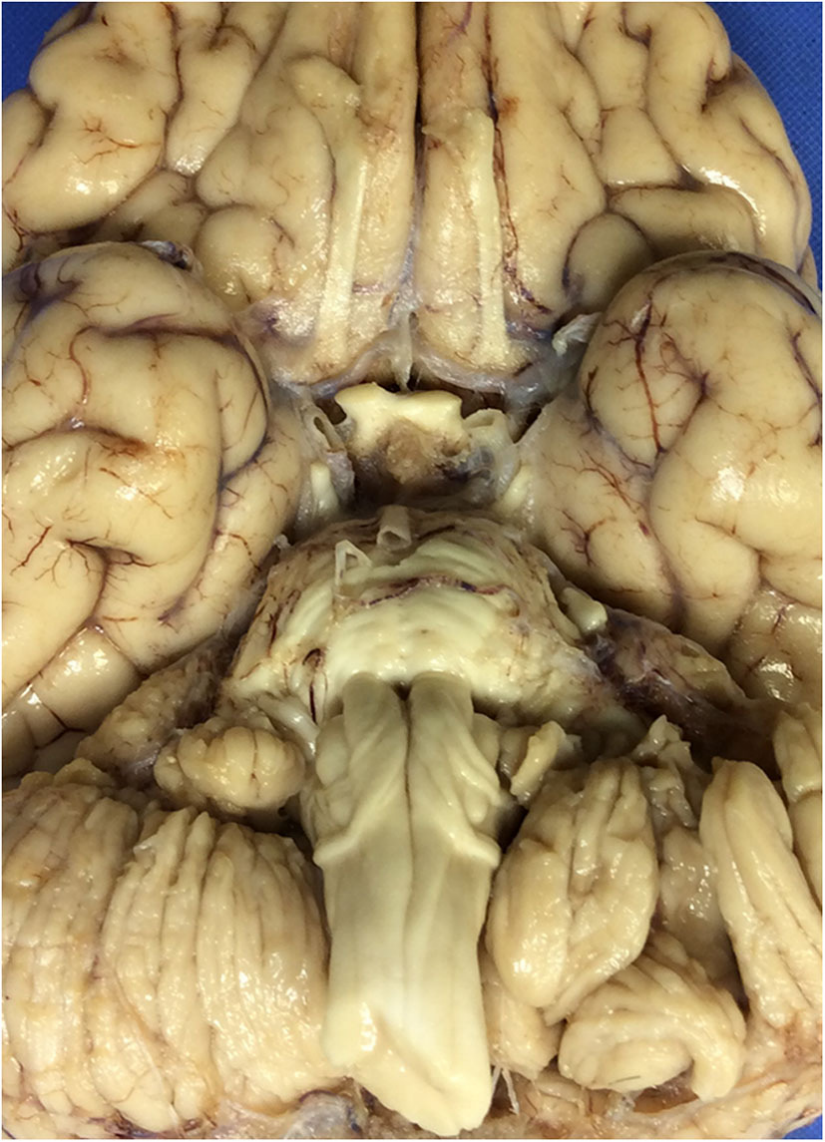
The ventral surface of the brain, including the ventral medulla in the index case. The leptominges and some of the cranial nerves rootlets have been removed. Note the presence of enlarged fiber bundles over the ventral surface of the medulla bilaterally.

At autopsy, the brain weighed 1,045 g and had a normal appearance for age. The cerebral vessels were intact and without atherosclerotic change. On the ventral surface of the medulla, there was an enlarged fiber bundle originating from the midline near the pontomedullary junction and extending inferolaterally to the lower edge of the olive (Figure 1), before turning superiorly toward the inferior cerebellar peduncle, the pathway of the normal AEAF. No other gross abnormalities were detected.

**Figure 2:**
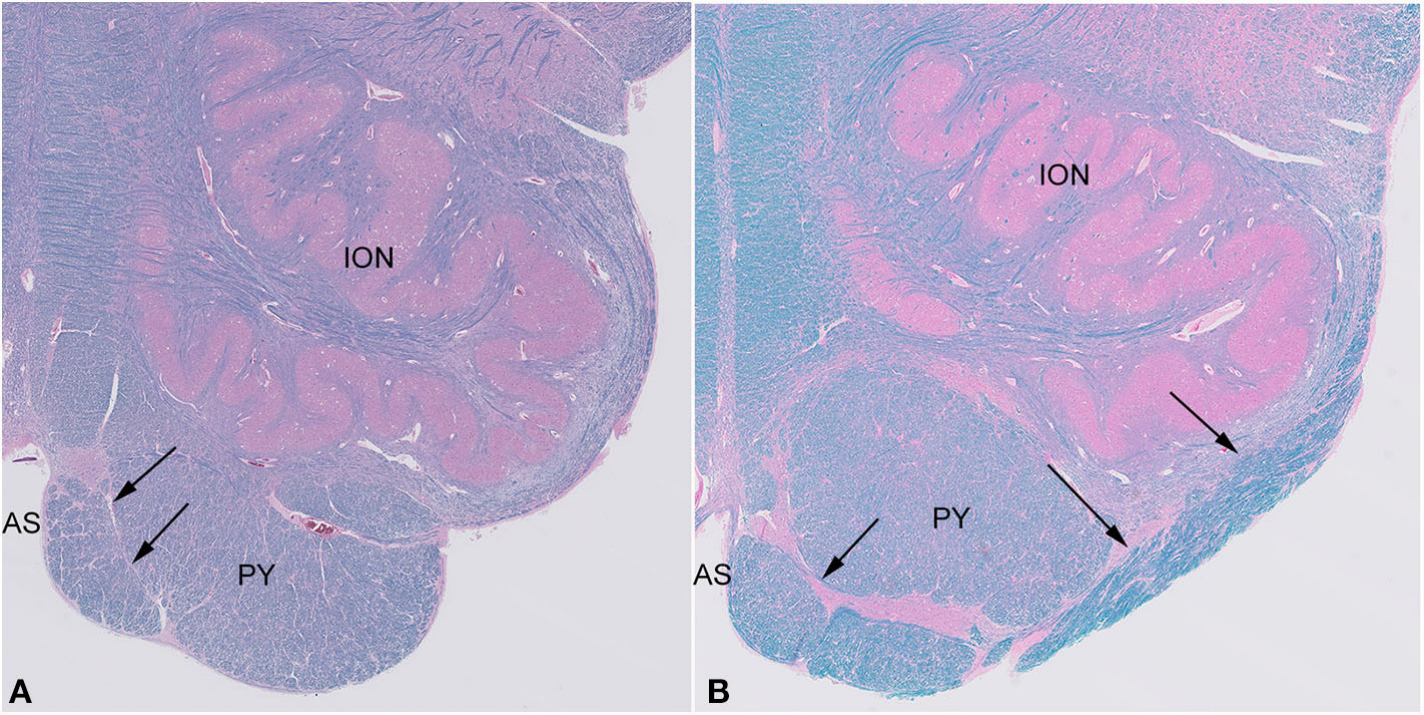
Sections of the medulla in the index case. The large fiber bundle is myelinated (blue color on LFB/H&E) and at a rostral level **(A)** is near the midline anterior sulcus (AS), medial and ventral to the pyramid (PY), while at a more caudal level **(B)**, it courses over the ventral surface of the pyramid (PY) and lateral surface of the olive (ION).

Histological examination confirmed the presence of large white matter tracts on the ventral surface of the medulla bilaterally (Figure 2). The fiber bundles were located medially in rostral sections (Figure 2A), and in more caudal sections, extended over the surfaces of the pyramids and laterally over the surfaces of the olives (Figure 2B). No other structural abnormalities were identified in the brain or brainstem. Other autopsy findings included cardiomegaly (787 g) with concentric left ventricular hypertrophy (1.9-cm wall thickness), focal myocardial entrapment of the left anterior descending coronary artery, coronary artery atherosclerosis (50% stenosis of the left anterior descending coronary artery), pulmonary intra-alveolar hemorrhage and congestion, hepatomegaly with multiple hemangiomas, bilateral glomerulosclerosis, leiomyomata uteri, and splenomegaly. The pathologic findings were thought to represent the complications of hypertension and atherosclerosis, with chronic cardiac ischemia and death due to a cardiac arrhythmia.

## Materials and Methods

Two adult patients with no significant neurologic history were studied as control subjects (age/sex: 76 years/male and 81 years/male). Postmortem intervals were 24 h (index case: 39 h, controls: 16 and 61 h). After fixation of the whole brain in 10% formalin for at least 14 days at room temperature (index case, 23 days, controls, 24 and 20 days), the brains were cut in the same manner as the index case, with ~1-cm-thick coronal sections of the cerebral hemispheres, 3-mm-thick transverse brainstem sections, and 5-mm-thick parasagittal sections of the cerebellum. The brainstem sections examined were the same level in the control cases and the index case.

Tissue blocks of ~3-mm thickness were prepared for histologic sections. These blocks in all cases included four levels of the medulla, from the most rostral to the most caudal levels of the ION, and all stains were performed on all levels of the medulla in both the index and control cases. The tissue blocks were embedded in paraffin, and histologic sections were cut at 8-μm thickness and stained with Luxol fast blue and hematoxylin–eosin (LFB/H&E) for preliminary analysis [0.1% Luxol fast blue, Harris hematoxylin solution (Sigma Diagnostics, cat. # HHS-32), and Eosin Y Alcoholic solution (Polyscientific, cat. # s2186-1gl)]. The modified Bielschowsky histochemical stain (Luna's method) was performed for examination of axonal density (Luna, 1968). Trichrome staining (Gomori's one step trichrome; Chromotrope 2R, light green FCF, in phosphotungstic acid with glacial acetic acid) was performed to delineate the fiber pathways as they approached the pial surface, and toluidine blue stain (toluidine blue; Polyscientific) was performed to highlight neurons and Nissl substance. Immunohistochemical stains for neurofilament and calbindin-2 (CALB2) (calretinin) were performed on sections cut at 4-μm thickness. Neurofilament immunohistochemistry was used to demonstrate neuronal cell density and axons (pre-diluted, mouse monoclonal, NF-L, clone 2F11, Dako Omnis Autostainer with a low pH pretreatment and Dako's Omnis Detection kit containing the FLEX HRP polymer, recognizing the low-molecular-weight subunit of neurofilaments, NEFL). An immunohistochemical stain for CALB2, the calcium-binding protein also known as calretinin, was used to identify CALB2-expressing neurons in the medulla (Dako, prediluted, mouse monoclonal, clone DAK-Calret 1, Dako Link 48 Autostainer with a high pH pretreatment, and Dako Link Detection kit containing the FLEX HRP polymere). Slides stained with LFB/H&E and Bielschowsky were scanned using a Philips Ultrafast 1.6 RUO whole-slide scanner. The ANs of both control cases and the index case were measured using Image Pro to determine cross-sectional area. The transverse area of the ANs was measured in all levels of the medulla, and the average transverse area calculated. The AEAF was measured using manual morphometry at the level of the upper medulla, just caudal to the pontomedullary junction, where it was located lateral to the anterior sulcus. The pyramid was also measured at this level. The axonal density within the AEAF and pyramids was measured using digital images, with manual quantification of axons. For the AEAF, only areas where bundles of axons were in cross-section were quantified.

## Results

**Figure 3:**
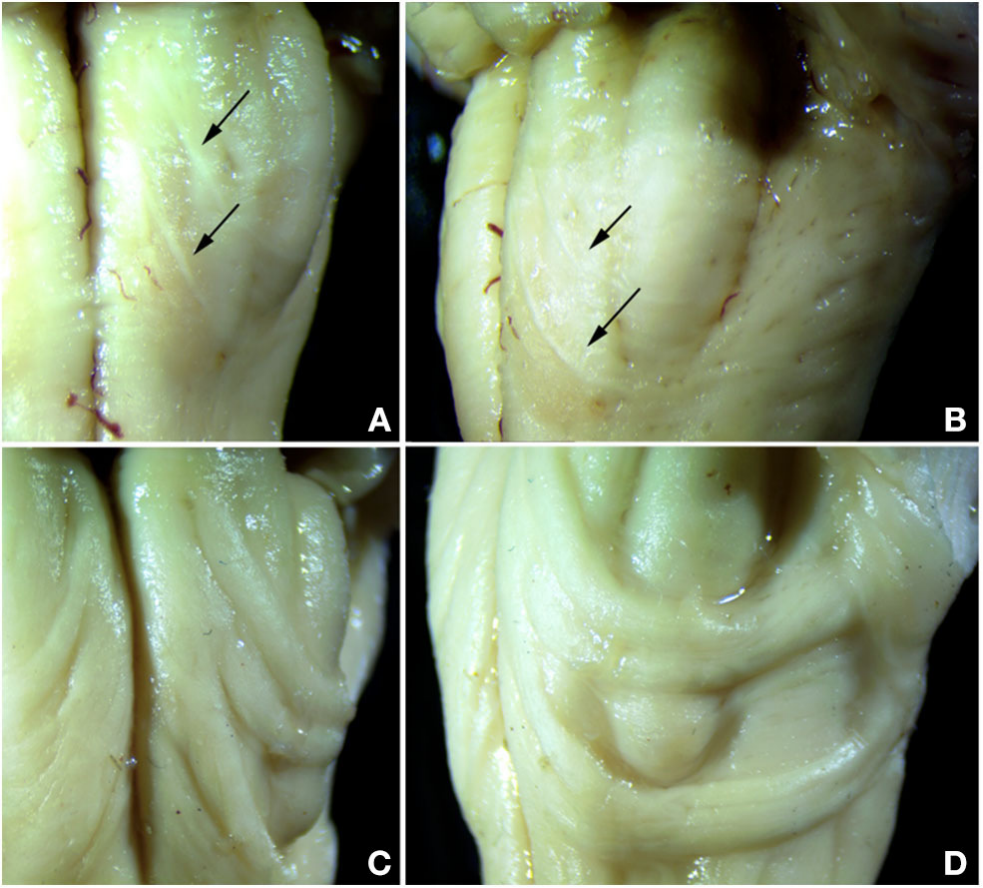
Comparison of normal AEAF (top panels) and the hypertrophic AEAF in the index case (lower panels) viewed from ventral (left panels) or lateral (right panels) aspect. The normal AEAF can be seen arising near the midline just caudal to the pontomedullary junction and looping down and around the inferior olive **(A,B)**. Two distinct small fiber bundles are visible, the caudal bundle passes immediately caudal to the olive, and the more rostral bundle crosses the olive over its lower portion. The AEAF in the index case **(C,D)** travels the same course, but was markedly enlarged.

**Figure 4:**
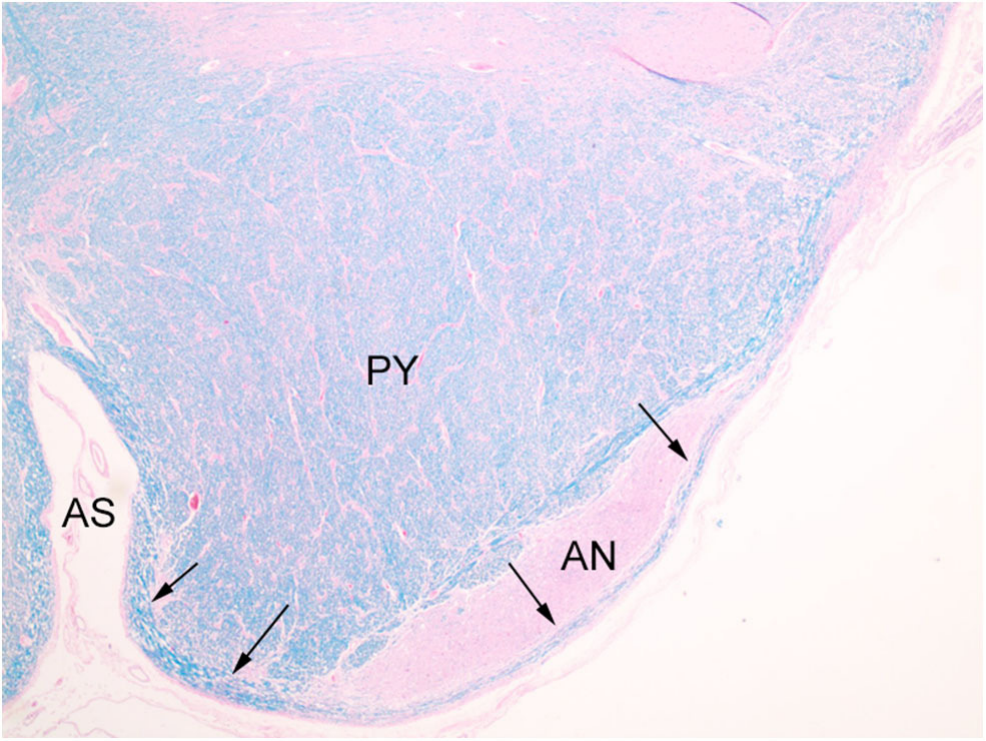
Normal AEAF (arrows), overlying lateral portion of the arcuate nucleus (AN). (LFB/H&E) The midline portion may be seen within the medullary anterior sulcus (AS), running parallel to plane of section along the surface medial to the pyramid (PY), and the lateral portion is seen on the surfaces of the pyramid and arcuate nucleus.

**Figure 5:**
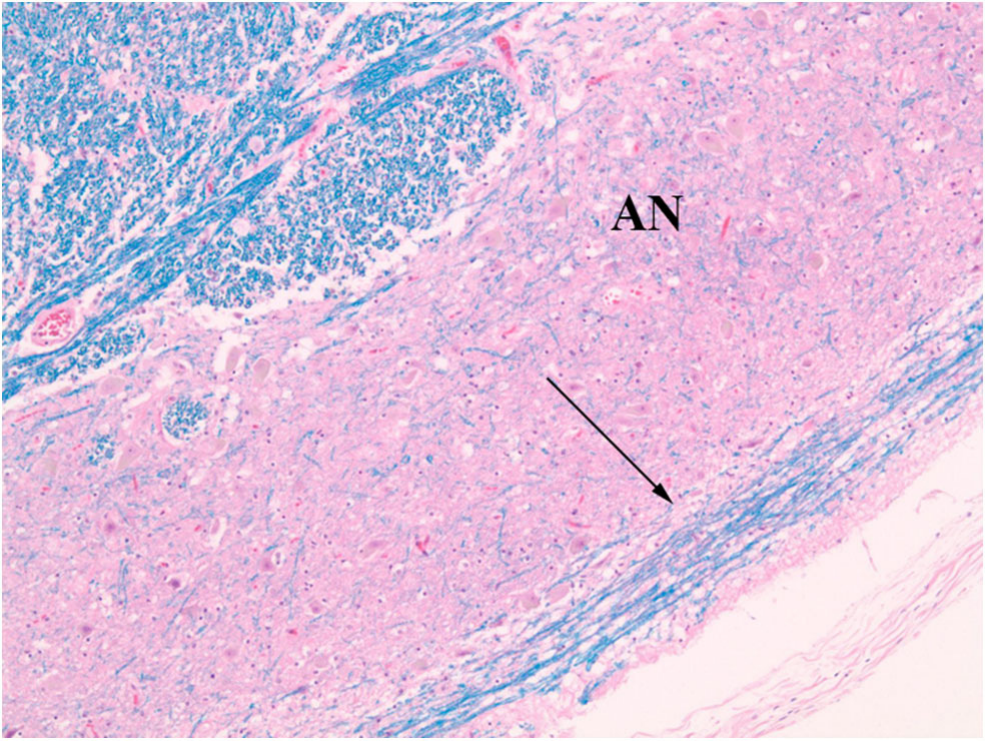
Normal AEAF (arrow). (LFB/H&E) The fiber bundle courses over the ventral surface of the arcuate nucleus (AN) at this level.

Both control cases showed the typical appearance of the AEAF, which is barely visible grossly but may be seen with a dissecting microscope (Figures 3A,B). It is composed of a small myelinated fiber tract (Figures 4, 5), with the most medial portion located just lateral to the anterior sulcus of the medulla. The medial portion is sheet-like and seen at all levels of the medulla; the lateral portion travels ventrally and laterally around the pyramid, the AN, and the olive. The lateral portion of the AEAF has two grossly visible branches, one coursing across the surface of the olive just below its midportion and the second coursing across the surface at the inferior edge of the olive (Figures 3A,B).

**Figure 6:**
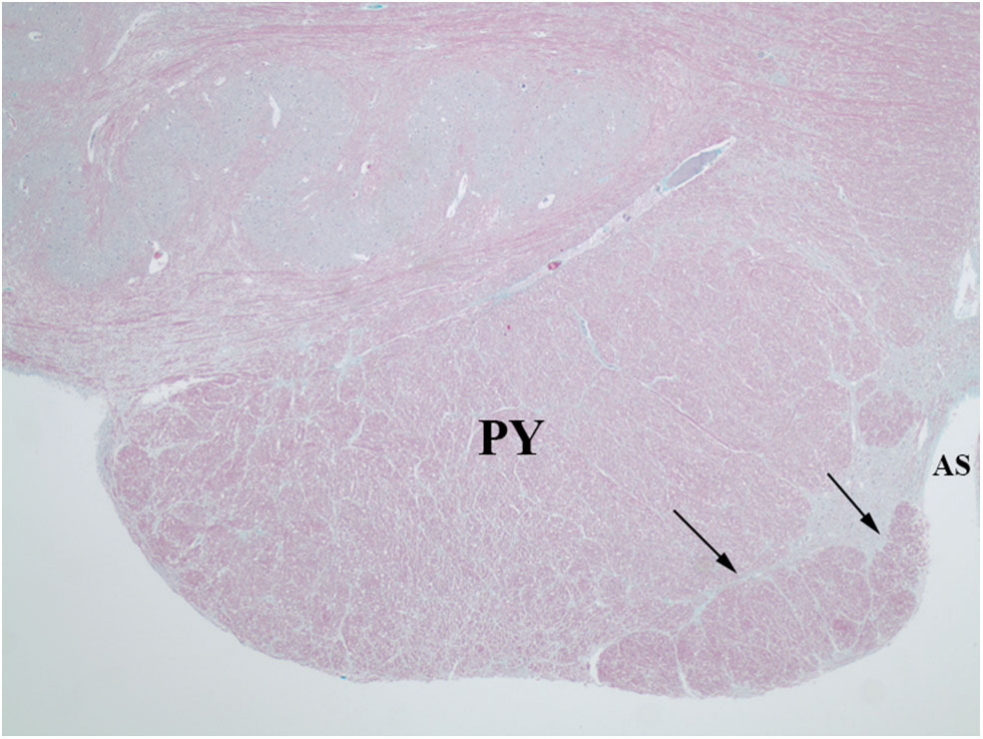
Trichrome stain highlights the delicate septum between the pyramid (PY) and the hypertrophic AEAF (arrows). The anterior sulcus (AS) of the medulla is on the right.

**Figure 7:**
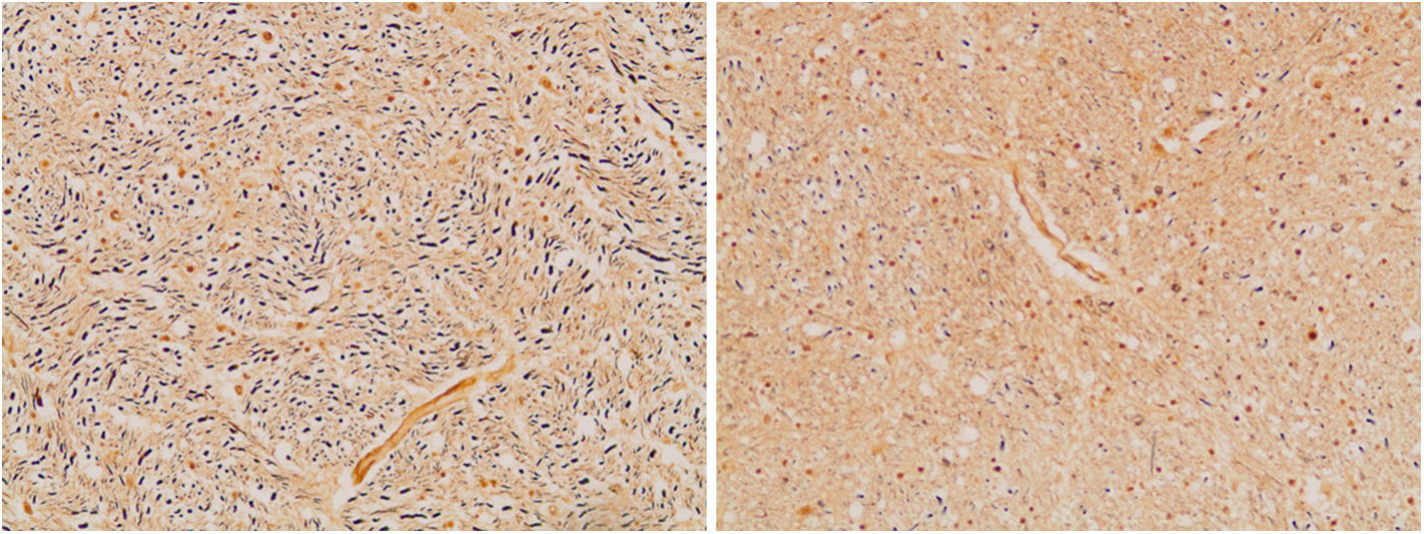
Axonal density in the medullary pyramid (left panel) and the AEAF (right panel). (Bielschowsky stain) The pyramid has a greater axonal density than the AEAF, making the two tracts easily distinguished on the silver stain.

The index case showed a large myelinated tract on the ventral surface of the brainstem (Figure 2). The pathways taken by these bundles corresponds exactly to the path of the normal AEAF, especially evident when compared grossly to control cases (Figures 3A–D), and as such, it is best viewed as hypertrophy of the AEAF. The tract was myelinated on LFB stains (Figure 2), with a faint septum separating it from the adjacent pyramid, highlighted on trichrome stain (Figure 6). The arcuate and inferior olivary nuclei appeared normal histologically. The density of neurons on Bielschowsky, toluidine blue, and neurofilament stains appeared the same as in the control cases. The axonal density of the AEAF appeared less than the axonal density in the medullary pyramid (Figure 7), both in the controls and index case.

Morphometric analysis of the AN showed an average cross-sectional area of 1.06 and 1.15 mm^2^ for the control cases and 1.01 mm^2^ in the index case. This is consistent with prior findings (Paradiso et al., 2018), which measured the cross-sectional areas of the normal ANs and found average areas of 2.76 ± 0.55 and 1.06 ± 0.3 mm^2^ in their cases.

The medullary pyramids, which appeared to be of normal size, had cross-sectional areas of 8.23 mm^2^ in the controls and 7.60 mm^2^ in the index case. The AEAF bundles in the control and index cases were 0.03 and 2.01 mm^2^, respectively, with imputed diameters of 0.18 and 1.60 mm, respectively. Using the calculated cross-sectional area of the AEAF and axonal densities estimated by Bielschowsky stain, we estimate that the normal lateral bundles of the AEAF contain ~450 axons, whereas in the index case, the hypertrophic AEAF contained ~18,000 axons. This hypertrophy represents a 40-fold increase in the number of fibers, 60-fold increase in cross-sectional area, and 9-fold increase in diameter.

**Figure 8:**
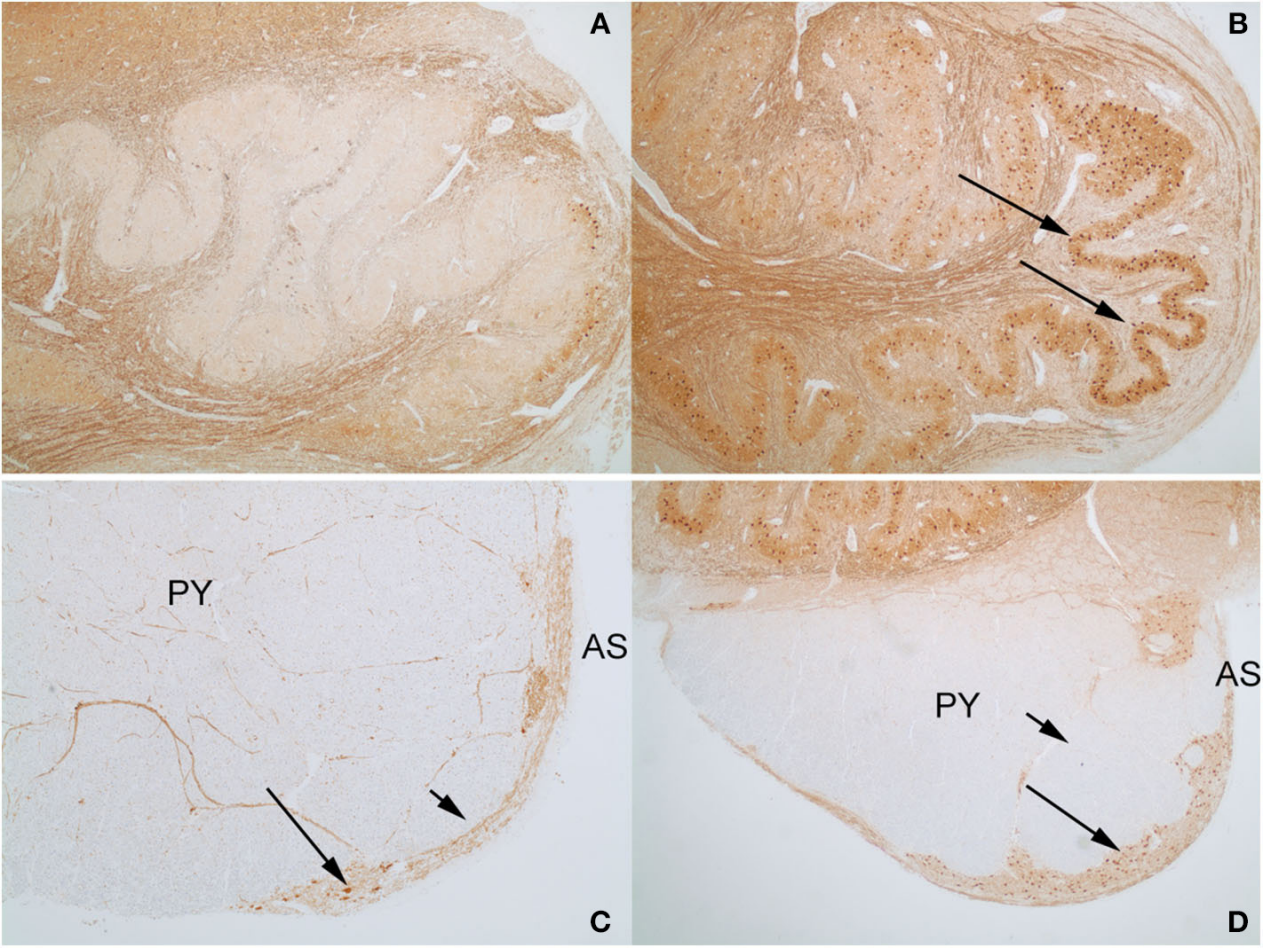
Calbindin-2 (calretinin; CALB2) expression in control case on the left **(A,C)** and index case on the right **(B,D)**. The normal AEAF contains calbindin-2 (**C**, short arrow), while the hypertrophic AEAF (**D**, short arrow) is largely negative for calbindin-2. The AN (**C,D**, long arrows) and the ventrolateral portion of the inferior olivary nucleus in the index case (**B**, long arrow) express calbindin-2 (IHC, calretinin). PY, pyramid; AS, anterior sulcus of the medulla.

An interesting difference between the two control cases and the index case was the expression pattern of CALB2 (calretinin). In both control cases and the index case, most of the large neurons in the AN expressed CALB2 intensely. The dorsal portion of the ION showed only a faint staining of the majority of neurons that was no greater than the background staining of neuropil (Figure 8A), but higher than the staining of endothelial cells. In contrast, there were strong CALB2-expressing neurons in the ventrolateral portion of the ION (Figure 8B), both in one control and in the index case. In both control cases, the AEAF had CALB2-positive axons, highlighting the pathway (Figure 8C); however, the hypertrophic AEAF was largely negative for CALB2 expression (Figure 8D).

## Discussion

We report a rare anatomic variant of a markedly enlarged AEAF on the ventral medullary surfaces bilaterally. The hypertrophic AEAF was nine times larger in diameter than normal, whereas AN and ION appeared histologically normal in size and neuronal distribution. Morphometric analysis of the AN confirmed that it was within the normal range previously reported (Paterson et al., 2009). Rare examples of unilateral hypertrophy of the AEAF have been described as circumolivary bundle enlargement (Swank, 1934; Williams, 1997; de Caro et al., 1998); however, bilateral hypertrophy of the AEAF has not been described previously, and its presence raises questions about the potential origin of the excess fibers of the AEAF. No aberrant fiber pathways were identified histologically within the medulla on H&E/LFB myelin stains or on axonal stains; no aberrant bundles of myelinated fibers entering the AEAF could be identified, and none could be identified emanating from the ION, the AN, or the pyramids. Calbindin-2 (CALB2, calretinin) expression was identified in the AN, as described previously (Fu and Watson, 2012; Baizer, 2014), but was also present in the ventrolateral portion of the ION, both in the index case and in one of the control cases. Although faint CALB2 staining has been reported within the ION (Baizer et al., 2018), the ventrolateral ION neurons in both the index case and one of the control cases were stained as intensely as the neurons in the AN. The fibers within the normal AEAF contained CALB2 in both controls, but the great majority of fibers within the hypertrophic AEAF did not.

Our findings are pertinent to the ongoing scientific interest in the development of the AN in humans. There have been two main theories on the development of the AN. It was originally proposed (Essick, 1912) that the AN neurons were related to neurons of the basis pontis based on their migration from rhombic lip neurons in development. It also had been recognized, however, that the rhombic lip is the origin of neurons not only of the basis pontis, but also the ION and AN (Essick, 1912; Rodriguez and Dymecki, 2000; Gilthorpe et al., 2002), that the AN and ION neurons begin migration prior to the pontine neurons, and that the neurons of the AN may bear a closer relationship to the ION neurons. Both ION and AN neurons migrate over the lateral and ventral surface of the medulla in a path that resembles the AEAF (Essick, 1912; Rasmussen and Peyton, 1946; Filiano and Kinney, 1992). Immunohistochemical analysis also has been used to study the origin of the AN in the mouse, which supports an origin related to the ION due to shared calbindin-1 (CALB1) and neurotransmitter expression patterns (Kinney et al., 2007; Fu and Watson, 2012; Baizer, 2014; Yu et al., 2014). In support of the idea that the AN is more closely related to the ION (Kinney et al., 2002, 2007; Fu and Watson, 2012; Yu et al., 2014), the neurons of the ION and AN both project to the cerebellum via the inferior cerebellar peduncle rather than the middle cerebellar peduncle, the ION and AN of the mouse express CALB1, the ION and AN have a gene signature pattern associated with climbing fibers, and Fu and Watson (Fu and Watson, 2012) have shown that clusters of neurons on the ventral surface, which they term the AN, develop predominantly from the inferior olive in the C57BL mouse.

Our findings in a patient with bilateral hypertrophic AEAF showed the AEAF was nine times the normal diameter in the presence of a normal size AN. The origin of the additional fibers was not identified; however, there was expression of CALB2 in both the AN and the ventrolateral ION. The similarity in CALB2 expression in the ventrolateral ION and the AN further supports the findings of Fu and Watson (Fu and Watson, 2012) that the AN may bear a relationship with the ventrolateral ION in the mouse. The fibers of the hypertrophic AEAF did not express CALB2, raising the possibility that the CALB2-negative neurons of the dorsal portion of the ION might have been a source of these fibers in this rare case (Fu and Watson, 2012; Yu et al., 2014). Alternative possibilities are that the fibers arose from another nucleus in the brainstem or may be related to a cerebropontocerebellar pathway.

## Conclusion

The AN of the medulla is of great interest because of its potential involvement in SUID and late fetal stillbirth (Filiano and Kinney, 1992; Matturri et al., 2000, 2002a,b; Rubens and Sarnat, 2013). Some studies have shown that infants dying of SUID have hypoplastic arcuate nuclei, and some adults dying of respiratory failure in other neurologic conditions may also have involvement of the AN (Tada et al., 2009). However, little is known of the arcuate's major efferent pathway, the AEAF. This is the first documentation of the normal size and axonal content of the AEAF and the first description of a case of bilateral hypertrophy of the AEAF. The source of the additional fibers within the hypertrophic AEAF could not be definitively identified; however, the absence of CALB2 expression in the fibers of the hypertrophic AEAF and the finding of CALB2 expression only in the ventrolateral portion of the ION and not in the dorsal portion of the ION raise the possibility that the dorsal ION may have contributed fibers to the AEAF in this case.

## Data Availability Statement

The raw data supporting the conclusions of this article will be made available by the authors, without undue reservation.

## Author Contributions

DA and RS identified the variant, designed the experimental plan, and analyzed the results. DA performed the manual morphometry, edited, and prepared the final version. RS analyzed data and wrote the first draft of the paper. RT performed the digital morphometry using Image Pro. TV processed all the tissue, performed all the histology and tissue level alignment, and edited the procedure portions of the manuscript. All authors reviewed the manuscript and figures.

## Conflict of Interest

The authors declare that the research was conducted in the absence of any commercial or financial relationships that could be construed as a potential conflict of interest.
